# Gazing at the partner in musical trios: a mobile eye-tracking study

**DOI:** 10.16910/jemr.11.2.6

**Published:** 2018-07-16

**Authors:** Sarah Vandemoortele, Kurt Feyaerts, Mark Reybrouck, Geert De Bièvre, Geert Brône, Thomas De Baets

**Affiliations:** LUCA School of Arts, Leuven, Belgium; KU – Leuven University, Belgium

**Keywords:** Ensemble playing, gaze direction, gazing at the partner, eye movements, mobile eye-tracking, musical trios, individual differences

## Abstract

Few investigations into the nonverbal communication in ensemble playing have focused on gaze behaviour up to now. In this study, the gaze behaviour of musicians playing in trios was recorded using the recently developed technique of mobile eye-tracking. Four trios (clarinet, violin, piano) were recorded while rehearsing and while playing several runs through the same musical fragment. The current article reports on an initial exploration of the data in which we describe how often gazing at the partner occurred. On the one hand, we aim to identify possible contrasting cases. On the other, we look for tendencies across the run-throughs. We discuss the quantified gaze behaviour in relation to the existing literature and the current research design.

## Introduction

The relationship between a wide range of aspects of ensemble playing and musicians’ gaze behaviour has recently gained more attention. This may be partly due to the realisation that bodily movement, a visual aspect of musical performance that has been studied extensively, must be attended to if it is to play a role in inter-performer communication. Yet observations regarding gaze as a communication channel in ensemble playing, whether as a means for gathering visual information on the partner or for cueing, are still scarce. The current literature that addresses gaze behaviour tends to do so anecdotally within the context of qualitative studies that describe gaze based on video recordings. However, researchers wishing to focus on musicians’ gaze behaviour in a relatively natural setting may consider making use of the recently developed technique of mobile eye-tracking.

The current paper reports on the initial results of such an undertaking and addresses methodological issues. The type of ensemble studied is the trio since this constellation combines the interactional richness of a group (as opposed to a duo) (9) with a minimum of complexities. Our research agenda is motivated by the aim to explain how musicians’ gazing at the partner may relate to their sense-making of the musical task. This means we eventually hope to relate gazing at the partner to the characteristics of the musical score and to the decision-making process during rehearsal. Thereby, we consider each individual musician a single case to be studied in depth, after which cross-case comparison will take place.

The current article reports on an initial exploration of a part of our data set. First, we describe how often gazing at the partner occurred to identify possible contrasting cases. Second, we compare the amount of partner-gazes across uninterrupted runs through the entire musical fragment in order to determine whether gazing at the partner increased or decreased. Our observations are based on a data set that shows four trio ensembles playing the same musical fragment, running through it four times each (sixteen run-throughs in total) in a rehearsal setting. The procedure also required the participants to work collaboratively on the musical fragment between the run-throughs (two times for half an hour), but data on these activities are not discussed here.

Below, we situate our research by presenting an overview of the main data collection methods used in studies that have addressed gaze behaviour in ensemble playing. We proceed by providing some technical insights into mobile eye-tracking. Last, we review results on gaze, as far as it relates to the musical task within ensemble playing. We note that gaze in performer-audience communication (see e.g. (2)), or in orchestral or choral conducting (see (36)), was deemed lying outside the scope of the current research.

## State of the art

The topic of gaze behaviour in ensemble playing has been illuminated by naturalistic and experimental research that employed data collection methods other than mobile eye-tracking. Gaze has been included in surveys on ensemble playing (8, 14, 33). A wide range of qualitative studies using video data, too, have addressed gaze as part of broader ensemble-related topics (11, 12, 17, 18, 26, 27, 41). Gaze has also been the focus of more detailed study, on the one hand by using video recordings of ensembles playing in natural settings (16, 21, 30), on the other by employing video cameras in experimental settings (22, 23, 32). Finally, some studies proceeded by employing several visual conditions, whereby gaze (at certain body parts or at the entire body) was either possible or obstructed (23, 24, 40). The existence of these studies indicates that the topic of gaze appears of interest to various researchers studying ensemble performance.

A particular challenge when using video data seems to be to avoid a trade-off between ecological validity and fine-grained gaze measurements. For example, some authors take head direction as a measurement for gaze direction, as is clearly stated in Moran (30) and Dardard et al. (10). The latter refers to Stiefelhagen (37), stating that head direction is sometimes a good approximation for gaze direction. Kawase (23) on the other hand, obtained the more fine-grained distinction between mutual gaze (gazing at each other’s body) and eye-contact (gazing into each other’s eyes) by means of an elaborate experimental design, using a screen between the musicians and a chinrest to fix their heads. Seen in this light, mobile eye-tracking can be considered an appropriate tool for measuring eye gaze in an interactional setting, as it allows a compromise between ecological validity and the need for measurements that capture the alternation between saccades (jerky movements from one target to another) and fixations (moments where the eyes remain relatively static and focused on the same target) (28).

Still, there are some limitations. First, we note that eye-tracking is not entirely new within the musical domain, as there is a growing body of research on music reading (see (29, 34)) in which various forms of video-based eye-tracking are used. In these studies, however, regardless of any methodological and technical varieties, the stimulus (in this case the musical score) is always presented as a stationary object (usually on a screen). When studying the eye movements of musicians playing in an interactional setting, visual targets are not known in advance and this calls for a different eye-tracking technique. Mobile eye-tracking (equally video-based) offers the advantage of allowing for a relatively naturalistic setting in which participants can direct their gaze at any point in space. In addition, they can move more freely in order to handle their instruments. On the downside, due to a relatively low sampling rate, gaze measurements require careful interpretation. High-accuracy eye-tracking systems collect data at up to 2000 Hz, whereas mobile eye trackers generally have a sampling rate of 60 Hz (1), although higher sampling rates are available as well. Since saccades may be shorter than 50 ms, Anantrasirichai et al. (1) argue that mobile eye trackers with a frame rate below 40 Hz may be inadequate to reliably distinguish between fixations and saccades.

Regardless of what kind of eye tracker is used, a second limitation is that the obtained data provide information about what lies in the participant’s central vision. Hence, peripheral vision, while it may play an important role in collaborative music making, cannot be studied. A third limitation, finally, concerns the occasional loss of data caused by the fact that the image of the video-recorded visual field is slightly smaller than the actual visual field. Therefore, the gaze cursor that moves across the video-recorded image of the visual field, thus indicating the point of regard, cannot be mapped onto the visual field image when participants look from the corner of their eyes.

To our knowledge, three studies thus far have incorporated mobile eye-tracking into the study of ensemble playing, other than our own pilot study (38, 39) and current research. Morgan et al. (31) devised a tool for real-time feedback on the body motion and eye gaze of an invisible co-performer employing eye-tracking headsets and small wireless accelerometers. Yamada et al. (43) tracked the gaze shifts of an expert and non-expert Japanese drum player playing together, calculating gaze shifts and percentages of time looking at the self, the opposite person, and other areas. An ongoing study by Bishop and Goebl (6) analyses mobile eye-tracking data, alongside motion capture and audio/MIDI data, from clarinet-piano duos to test whether visual communication between performers facilitates coordination and how. The duos performed three run-throughs, at the start, middle, and end of a rehearsal, followed by a run-through during which musicians’ views of each other were obscured. The use of several run-throughs renders their research somewhat comparable to our own pilot and current study, the design of which we explain in the next section.

Most studies with results on gaze have dealt with aspects of the musical task, whether as a task set by the musical score or by the demands of ensemble performance. Some studies reveal that certain moments in the score can indeed be said to bear a relationship with gaze behaviour. Davidson (11) who video-recorded clarinet-flute duos observed that gazes at the partner do not happen regularly, but rather at major boundaries (the start and the end of the work and section endings). Furthermore, Williamon and Davidson (41), p. 61, state that the proportion of “direct, simultaneous eye-contact” (out of the total amount) increased across the two rehearsals, and public performance, at places in the score that were identified by the musicians (a piano duo) as important for coordination.

Results in other studies point out that gaze behaviour may also relate to aspects of the sounding performance. For instance, the topic of coordination was also addressed by measuring timing lags between musicians’ note onsets. Morgan et al. (32) and Vera et al. (40) concluded that gazing at the partner enhanced synchronisation. Furthermore, Kawase (23) studied piano duos and found that mutual gazing, but not eye-contact, enhanced synchronisation at tempo changes. In Keller and Appel (24), visual contact was found to cause a higher variability of key stroke asynchronies between the two pianists than in the condition where performers could not see each other, although ensemble coordination was not affected markedly. The authors suggest that visual contact could have encouraged the performers to be more expressive with the timing. In a second experiment by Kawase (23) it was suggested that gazing itself provided some coordination cues, although movement cues were necessary for strict coordination. This is not necessarily in contradiction with Keller and Appel’s (24) finding that the absence of visual contact did not affect coordination markedly, since the musical materials in their study contained no tempo changes and maintained a continuous metrical pulse (in contrast with Kawase’s study).

Another performance aspect that gaze has been shown to be related to is the relationship between leaders/soloists and followers/accompanists, as can be learnt from Moran (30) and Kawase (22). In both studies, accompanists looked at soloists longer than vice versa. As Kawase remarks, this shows that similar mechanisms regarding gaze may be at work in musical interaction (when considering melody allocation or leadership allocation) as in spoken interaction (when considering social status). For example, participants in a study by Foulsham et al. (15) looked more frequently and longer at high-status individuals than at low-status individuals when watching a clip of a group decision-making task.

Our own study tracked the gaze behaviour of musicians playing in trios consisting of a clarinet, violin and piano using Tobii Pro Glasses 2 (sampling rate 50 Hz). Gazing at the partner will eventually be related to the characteristics of the musical score and to the decision-making process during rehearsal via in-depth study of each individual musician. The aim of the current article, however, is to report on an initial exploration of a part of the eye-tracked data set, namely the four run-throughs each of the four trios played. First, we describe how often gazing at the partner occurred to identify possible contrasting cases. As studies on conversational interaction show, gaze behaviour is not only a means to an end, for instance to regulate turn-taking in unscripted conversation, but it is also a learnt behaviour shaped by social norms. As such, people tend to have an idea of what constitutes ordinary or deviant gaze behaviour (35). Not considering that gazing at the partner may be generally recommended in certain musical situations (i.e. at tempo changes), it may be hard to define what constitutes ordinary or deviant gaze behaviour in musical interaction. Indeed, the lack of clear norms regarding gaze behaviour presents musicians with the opportunity to display themselves as various sorts of artistic personae and allows them to actively engage with the audience (as was the case with *The Corrs* according to a study by Kurosawa and Davidson (27)) or to purposely ignore them. Given this flexibility and given the additional fact that, in conversations, individuals’ amount of gazing at the partner has been shown to differ substantially (25), we expect that the number of gazes at the partner in our study will differ regardless of the musical instrument of the participant.

Second, we compare the amount of partner-gazes across run-throughs in order to determine whether gazing at the partner increased or decreased. Williamon and Davidson (41) found that eye-contact increasingly occurred at places in the score that were identified by the musicians as important for coordination. The increase was found after comparing two rehearsals and a performance, i.e. three stages of different duration. As the authors suggest, gaze may have started to function increasingly as a coordinating device over the course of the rehearsal process, however the increase may also have been supported by a growing ease to look up from the score. Both the study by Williamon and Davidson (41) and our own study deal with interactions between unfamiliar musicians rehearsing unfamiliar music. The current design differs in that it allows for a comparison between runs through the same fragment, all in a rehearsal setting, thus providing the opportunity to determine whether a tendency can be detected regardless of any links to specific moments in the score.

## Methods

### Participants

Our data set consists of twelve musicians, who follow higher music education in Belgium and agreed to participate in an eye-tracking experiment. Except one, all study at LUCA School of Arts and were selected on the basis of their musical abilities as judged by the chamber music coordinator of the institution. One participant was found through social media and studies at the Royal Conservatory of Brussels. Four clarinet-violin-piano trios were formed making sure that no musician had ever played chamber music with any of the partners before. Two of the trios were all female, one included a male musician, and one included two male musicians. Ages ranged from 18–28 years (Mean age = 23 years). None of the musicians had ever played the composition chosen for the recording session. They consented in writing to taking part in the study and to the use of still images and audiovisual recordings for scientific purposes. At the end of the session each musician received a voucher.

### Setting, apparatus, stimuli

**Figure 1.**
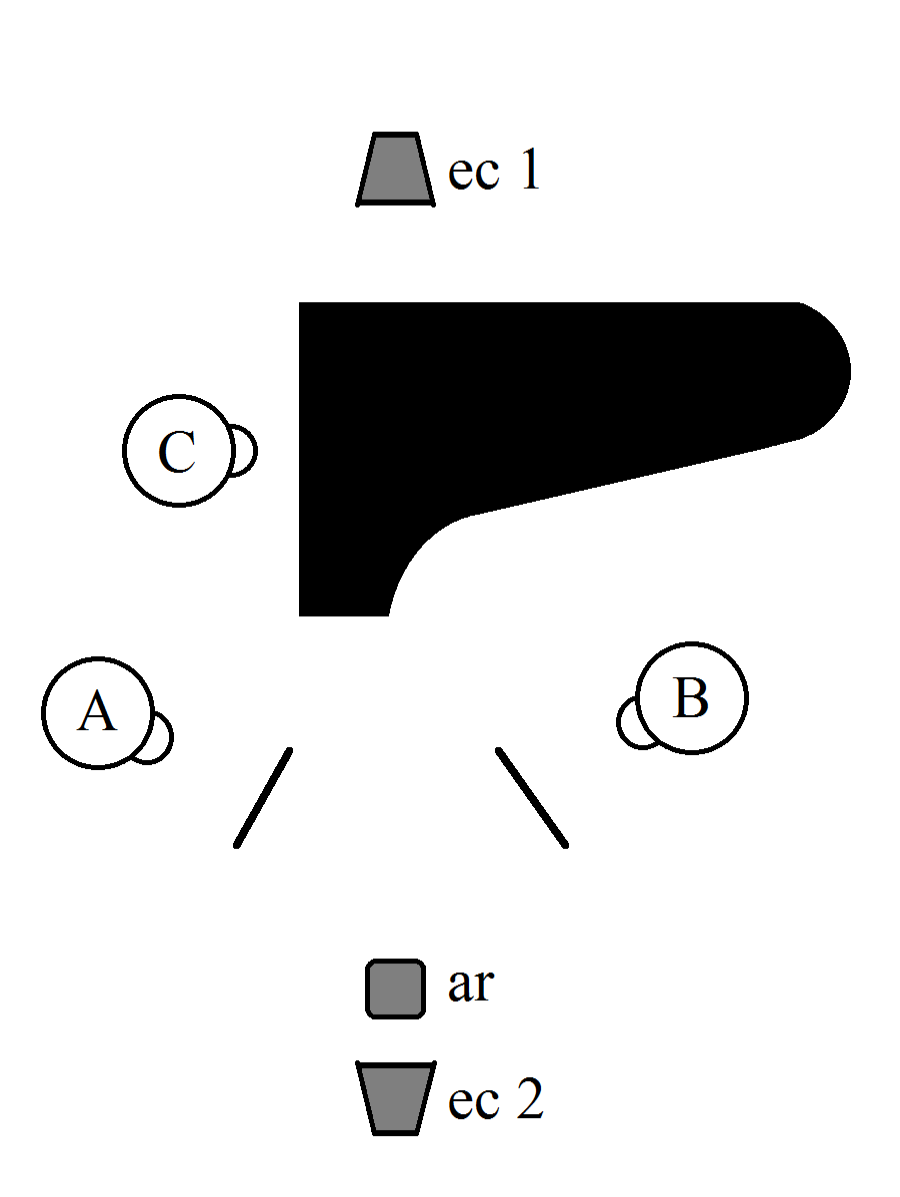
Plan of the set-up, indicating the positions of the violinist (A), clarinetist (B), pianist (C), external camera on the balcony (ec1), external camera in the seating area (ec2) and audio recorder on the front row of the seating area (ar). The large black object represents the grand piano, while the two black lines indicate the music stands of the violinist and clarinetist.

The recording session took place in the concert hall of the institution. The musicians were positioned the way they would be in a natural condition (Fig. 1), i.e. the clarinetist stood inside the wing of the piano, while the violinist stood next to where the pianist was seated. Clarinetist and violinist were faced toward each other and the height of their music stands was lowered just enough so that the participants could see each other’s head. The distance between the musicians was such that gazes at different large areas of the body could be distinguished (i.e., head, torso, legs, arms…). Smaller parts (for instance, eyes and mouth) could not be detected separately.

Each musician in the trio wore a binocular mobile eye tracker (Tobii Pro Glasses 2, sampling rate 50 Hz). For those who normally wore prescription glasses the eye tracker was fitted with lenses with approximately the same strength as the participants’ own. Two external cameras (frame rate 50 fps) captured the overall interaction. One was positioned in the seating area of the hall, filming a frontal view of the trio; the other filmed the back of the trio from the balcony above the stage. An audio recorder (TASCAM DR-2d) was placed on one of the front seats and guaranteed a reasonable sound quality.

The musical excerpt was taken from the last movement of Milhaud’s *Suite* for violin, clarinet and piano (measure 1 to 103 of the *Vif*-section). The musical parts carried a metronome marking of 120 bpm. At this speed the excerpt lasts about 2 minutes in performance. The marking could inform the participants about the envisaged performance tempo, however the researchers gave no instructions as to what tempo was expected. The musicians were also told not to use a metronome. The music was deemed appropriate for the study of individual differences and interactional dynamics, as the instruments are treated more or less as equal partners through an almost equal share in the melody and through passages that combine the melody with countermelodic material, rather than accompaniment patterns.

### Procedure

Upon arrival at the concert hall, the participants were briefly introduced to each other. The researcher explained the schedule for the session, handed over the musical parts and guided the clarinetist and violinist to an individual practice room. The pianist remained in the concert hall. The musicians were allowed to practice for half an hour, after which they were assembled for the eye-tracked rehearsal. The rehearsal followed a pre-determined schedule that alternated between uninterrupted runs through the musical fragment and rehearsal periods during which the participants were expected to work collaboratively on the fragment. The schedule was organised as follows: rehearsal period 1 (30’) – run-through – rehearsal period 2 (30’) – run-through – run-through. Except for the individual practice, the entire session was recorded with mobile eye trackers, cameras, and audio device. The eye trackers were recalibrated before each run-through. At the very end, each participant filled out a post-performance questionnaire. On the one hand, the questionnaire obtained information about the participants’ experience of the equipment and procedure. On the other, it aimed to collect possible data points for analysis by enquiring about the difficulties in the musical excerpt and by asking where in the score participants thought they had looked at a partner. Gaze will be analysed in the light of these responses at a later stage of our research. It is well worth noting that all participants, with one exception, considered the amount of individual practice time either adequate or too long. Regarding the amount of rehearsal time, participants stated either that no additional time was needed or that additional rehearsing on another day could be useful if they were to study the full musical piece. It therefore seems that the musical excerpt was not too difficult for the participants. The questions were formulated in Dutch and English. However, quite a few participants were not native English or Dutch speakers. Hence, after participants completed the form a brief one-to-one discussion followed with the researcher, mostly to ensure that the questions and answers were clear to both participant and researcher.

### Annotation of gaze behaviour

Prior to annotation, the gaze data of each trio member were exported as video files. These were synchronised with each other, with one of the external camera recordings and the audio recording in Adobe Premier Pro (but only the eye-tracked data and audio recording are of importance for the current publication). Synchronisation was enabled through the claps that were executed at the beginning and end of each run-through and rehearsal period. In the resulting quadvid, the audio of the eye-tracking videos and the external camera recording was disabled, leaving only the sound from the audio recorder. The synchronised data were exported at 25 fps and imported into the editing tool ELAN (42) to be annotated manually. The procedure thus far followed that of researchers in conversation analysis (see for instance (19, 20)).

Whenever the gaze cursor approached one of the partners, the annotator checked the location of the cursor frame by frame in order to determine the start and end of a partner-gaze. Partner-gazes were annotated as such when the gaze cursor fell onto the partner, including (parts of) the instrument, i.e. the clarinet, the violin and bow, and the keys of the piano. The annotated data set did not include moments where the gaze cursor fell *near* the partner. We will investigate these moments separately (in a later stage of our research), since these moments were clearly marked by a gaze shift toward the partner and therefore could be relevant for the study of gaze in ensemble interaction. Equally excluded from the annotated data set were 20 instances where it was unclear whether the gaze cursor pointed toward the partner due to overlap. These cases concerned some of the violinists. Due to the particular posture of violinists (who hold the instrument at the left side of the body) and their particular position in the trio (at the right side of the pianist), it was at times hard to distinguish gazes at the scroll of the violin and the violinist’s left hand from gazes at the pianist, who was seated ‘behind’ the scroll and left hand.

### Annotation of the sounding music

Musical bars were annotated manually on an additional tier in ELAN by listening to the audio recording. This included checking each bar in an initial annotation in order to eliminate traces of sound that belonged to the previous bar. Since ensemble playing always involves some asynchrony, this means that in our annotations the new bar started once all three musicians had arrived there. We note that asynchronies were overall hard to detect by ear so, for our research purposes, this procedure seemed adequate.

After analysis of the score, score characteristics were annotated on additional tiers, using the audio recordings, in a similar fashion as described above. The score analysis identified structural features (e.g. section endings, phrase endings, and smaller phrase segments), role allocation (indicating which instrument held the melody, countermelody, or accompaniment), role switches (moments where role allocation changed), rests, entrances and exits.

## Results

The analysis we report here is based on only the run-throughs, not the rehearsal periods, which will be analysed in a next stage of the project. Accidentally, all trios started their first rehearsal period with a complete run-through. This enabled us to compare four run-throughs across trios (one spontaneous run-through and three that were demanded by the researchers). They can be situated within the rehearsal schedule as follows: run-through (1) as part of rehearsal period 1 – remainder of rehearsal period 1 – run-through (2) – rehearsal period 2 – run-through (3) – run-through (4).

The musical fragment contained 103 bars of music. As was observed in our pilot study (39), playing always starts after a mutual gaze and very often finishes with a cluster of gazes at, and after, the end of the musical piece. This points towards two gaze situations that are different from the situation where one is in the midst of playing. Similar observations were made in the current data set. Since we did not want to smooth out differences between individual musicians during playing, the last bar was eliminated from analysis, as were gazes before the start of the music.

### Individual musicians’ amount of gazing at the partner

**Figure 2.**
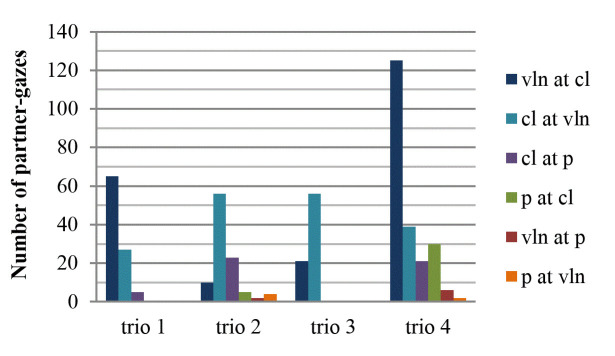
Number of partner-gazes for all four run-throughs in total. Partner-gazes take place from violin to clarinet (vln at cl), from clarinet to violin (cl at vln), from clarinet to piano (cl at p), from piano to clarinet (p at cl), from violin to piano (vln at p), and from piano to violin (p at vln).

Gazing at the partner in a trio constellation can occur in six directions, in this particular study between violin and clarinet, between clarinet and piano, and between violin and piano, each time in two directions. As regards the number of partner-gazes in all four run-throughs in total (Fig. 2), gazes occurred in both directions between violin and clarinet in all trios. However, in trios 1 and 4 the violinist looked more often at the clarinetist than vice versa. In trios 2 and 3, the clarinetist looked more often at the violinist than vice versa. Regardless of the specific gaze direction, in each trio the highest amount of partner-gazes happened between the violinist and the clarinetist.

Much less gazing at the partner can be seen in the interactions that involve the pianist. In trio 3, no one looked at the pianist and the pianist did not look at anyone. To be sure that, indeed, there was no visual interaction at all with the pianist in this trio, we checked whether gazes near (as opposed to *on*) the partner occurred. This was not the case. In trio 1, the clarinetist only looked five times at the pianist across all run-throughs. The pianist, again, did not look at anyone (nor gaze near the partner). There were no gazes at the pianist by the violinist (although overlaps occurred four times). In trios 2 and 4, gazes happened in all six directions. In both trios, gazes between violinist and pianist occurred only sporadically. Compared to that, the pianist received more than sporadic visual attention from the clarinetist in trio 2. In trio 4, both pianist and clarinetist looked more than sporadically at each other.

### Distribution of partner-gazes across run-throughs

**Figure 3.**
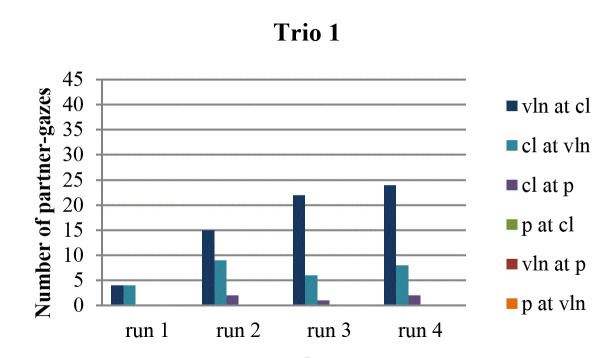
Number of partner-gazes across run-throughs in trio 1.

**Figure 4.**
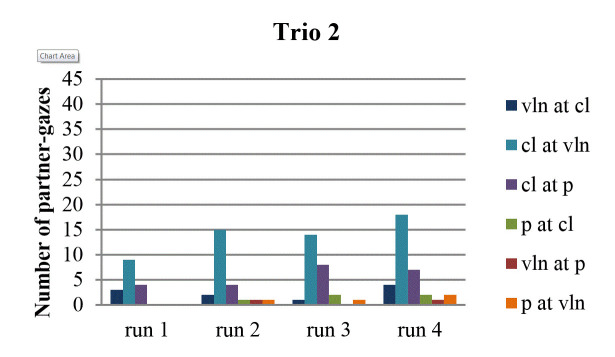
Number of partner-gazes across run-throughs in trio 2.

**Figure 5.**
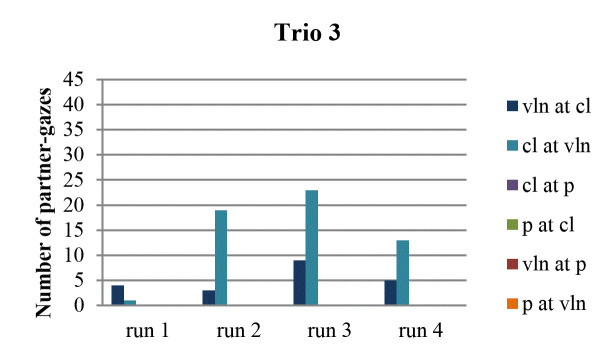
Number of partner-gazes across run-throughs in trio 3.

**Figure 6.**
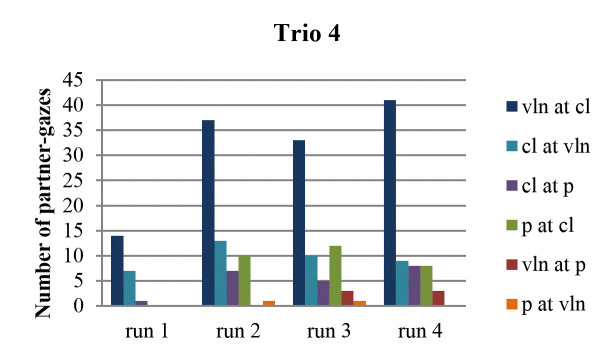
Number of partner-gazes across run-throughs in trio 4.

When we view each run-through separately, we see that gazing at the partner occurred least often in the first run-through (Fig. 3, 4, 5, 6). In terms of the amount of gaze directions that were represented in this first run-through, most trios (1, 2 and 4) reveal that gazes occurred in fewer directions than in the other run-throughs. Also, pianists never looked at anyone during the first run-through. As is the case with the amount of gaze directions, the first run-through tended to contain a lesser amount of partner-gazes than the other run-throughs. More specifically, this was the case in trio 1 (in all directions), in trio 2 (in all directions, except from violin to clarinet), in trio 3 (except from violin to clarinet), and in trio 4 (in all directions).

While still attending to each gaze direction within each trio, we consider the possibility of a tendency across all four run-throughs. Some instances of the last run-through contain the highest number of partner-gazes, namely in trio 1 (from violin to clarinet), trio 2 (from clarinet to violin), and trio 4 (from violin to clarinet). A steady increase of partner-gazes across run-throughs can be observed in trio 1 (from violin to clarinet) and – if one considers the total amount of gazes by a musician to both partners – in trio 2 (by the clarinetist and by the pianist). Finally, there are no musicians, whose gazing at the partner decreases across run-throughs and there are no musicians either, whose last run-through reveals the lowest number of partner-gazes.

## Discussion

### Individual musicians’ amount of gazing at the partner

As regards the amount of partner-gazes for all run-throughs in total, it was found that most visual interaction occurred between clarinetists and violinists, some between clarinetists and pianists, and no or only sporadic visual interaction between violinists and pianists. A possible explanation could lie in the particular musical excerpt used. Although the three instrumentalists overall are treated as equal partners (through an almost equal share in playing the melody and through passages that are polyphonic in nature), exchanges of the melody happen at a quicker pace between the violinist and clarinetist than with the pianist. According to our analysis, the violinist and clarinetist take over the melody each 12 times, whereas the pianist does so only 4 times but for a longer stretch of time.

Another plausible interpretation relates to the (natural) set-up of the musicians. Violinists and clarinetists only have to look up from the score in order to see each other, whereas a slight turn of the head to the right is needed for the clarinetists and pianists, and a turn of the body for the violinists and pianists. The same remark applies to the study by King and Ginsborg (26), where (relatively) little observable gazing at the partner was found. In their study a head turn was necessary for the singers and pianists to see each other within central vision, similarly to the clarinet-piano interaction in our study. While a common explanation for both studies might be that pianists do not often look at their partners, the set-up was insufficiently accounted for in order to enable a meaningful interpretation of the frequency of partner-gazes.

While the musical fragment and the set-up in our study may have caused differences between instrumentalists within the trio constellation, a comparison across trios seems to defy attempts at generalisation, confirming our expectation that differences in gaze behaviour would occur regardless of the specific instrument played. Between clarinetists and violinists, both the scenario of clarinetists looking more often at violinists (Trios 2 and 3) and the opposite scenario (Trios 1 and 4) occurred. In addition, the absolute frequency of gazing at the partner differed widely: In trio 2 the violinist only looked 10 times at the clarinetist, whereas the violinist in trio 4 did so 125 times. Also, while in some trios gaze occurred in all six directions (Trios 2 and 4), in other trios certain gaze directions were not represented in any of the four run-throughs. In extreme cases, the pianist was not looked at by anyone and/or did not look at anyone. Individuals thus differed to the extent that they looked at both partners, only looked at one partner and ignored another, or never even used (foveal) gaze as a means of communication.

As for the pianists’ infrequent looking at the partner, the need for seeing the keys may have to be taken into account. However, the pianists reported only few technical difficulties that required close visual attention. Pianists 1 and 3 reported a jump and a glissando. Pianist 2 indicated only the glissando and pianist 4 did not report any technical difficulties. Pianist 3 pointed out additional difficulties that required looking at the keyboard in two passages, each six bars long. We also note that they may have experienced a higher need for reading the score than the other musicians because of having to read two staves, but our self-reports did not cover this issue.

A substantial difference as to how often musicians looked at their partner can also be seen in Biasutti et al. (5). In daily conversations, too, participants’ amount of gazing at the partner may differ substantially. Specifically, Kendon (25) found that the amount of time spent gazing at the partner varied from 28% to over 70% of the total duration of the analysed samples. We note that, in our data set, the total amount of time spent looking at the partner was much less, as can be expected since the musicians also had a reading task to fulfill, and varied between 0% and approximately 15% of the entire duration of a run-through. As for the musicians who did not look at all at their partner (pianists 1 and 3), their situation mirrors the experimental conditions in Keller and Appel (24) and Kawase (23), whereby musicians could not see each other. The former study, using a fragment without metrical changes, found no remarkable effect of visual conditions on ensemble coordination, while in Kawase’s study there was an effect on the coordination of tempo changes. In line with Keller and Appel’s (24) study, our musical fragment did not contain tempo changes or changes in metrical pulse. Consequently, it may be that pianists 1 and 3 did not consider gazing at the partner, at least via central vision, necessary for the sake of temporal coordination. In fact, when listening to the audio, no disturbing asynchronies could be heard in any of their trio’s performances (as was the case for all trios). Thus, the musical sounds themselves may have provided them with the necessary cues to synchronise. Evidence from a study by Vera et al. (40) could support such an argument. While other studies have mentioned synchronisation and musical coordination as a possible role of gazing at the partner (11, 12, 21, 32, 41), this functionality has to be considered with respect to the musical characteristics and to competing coordination strategies that individuals may draw on to different extents.

### Distribution of partner-gazes across run-throughs

We also looked at the distribution of partner-gazes across run-throughs to see whether a tendency could be observed. In the first run-through, partner-gazes were fewer and represented less gaze directions than in the other run-throughs. Since at this moment the musicians were still unfamiliar with each other and with the overall sound of the musical fragment, familiarity may well be a good explanation for the frequency of partner-gaze. Once the newness of the music and the social pressure to perform well in front of unfamiliar partners have been overcome, musicians are better able to let go of the notes on the score. Also, additional rehearsing after individual practice may have contributed to a diminished/diminishing need for close note-to-note reading in run-throughs 2, 3, and 4. Alternatively, the first run-through was not an ‘official’ one in the sense that it was not requested by the researcher. As it was an inherent part of the rehearsal period, there may have been less pressure on the participants to perform well, causing a difference in their gaze behaviour. Last, we note that the first run-through was played slower than the other run-throughs by trios 2 and 3, causing the conditions in which gaze occurred to be slightly different in the first run-through for those trios. (Specifically, all trios played run-throughs 2, 3 and 4 at performance tempo, with the total playing time varying between 1’45” and 1’55”. Trios 1 and 4 stayed within this range for the first run-through, while in trios 2 and 3 the total playing time was 2’51” and 2’20” respectively.)

A tendency across all run-throughs was otherwise hard to find. The last run-through was found to contain the most partner-gazes in the case of a few musicians, while sometimes the amount of partner-gazes also increased across all run-throughs. The opposite – the last run-through containing the least partner-gazes or a decrease across run-throughs – was not found. Although this is little evidence for a general tendency, this somewhat confirms Williamon and Davidson’s (41) suggestion that the increase of eye-contact at important momments for coordination should not be explained solely by a tendency for gaze to increasingly function as a coordinating device. By contrast, in our pilot study (39), no tendency for partner-gaze to occur more frequently could be found in any of the three duos that were analysed. This may be due to the fact that the four run-throughs in the pilot study took place on two different days and constituted ‘snapshots’ in the middle of a rehearsal process, which was initiated by the musicians themselves before they were asked to take part in the study. The tendencies found in the current study may thus be typical for a very first rehearsal when performers do not know each other and try out a new piece for the first time.

## Conclusions

In this exploratory paper, we investigated the amount of gazing at the partner in four trios (clarinet, violin and piano) where musicians were unfamiliar with each other and with the musical fragment. Their gaze behaviour was recorded with mobile eye trackers in a rehearsal context in which they played four run-throughs. As the gaze frequencies within this particular trio constellation could not easily be interpreted, follow-up research may benefit from enquiring into matters relating to the set-up of the musicians and the choice of the musical fragment. Yet, our results indicate that individual musicians’ amount of gazes at the partner may differ substantially regardless of their instrument. Also, while gazing at the partner occurred much less during a first run-through prior to any collaborative rehearsing, a tendency across all run-throughs was harder to detect for the remainder of the session and, only in the case of a few musicians, was an increase of partner-gazes found.

## Future analysis and research

The current analysis focused on the direction in which gazing at the partner occurred. Further analysis may delve deeper into the interactional patterning of gaze by the three musical partners. For instance, a gaze at the partner may or may not be returned by the addressee. As regards this patterning, Kawase’s (22) terminology may be useful, as he distinguishes between “mutual gaze” (gazing at the partner’s body) and the sub-category “eye-contact” (looking into each other’s eyes). Some authors fail to do so and use the term eye-contact without clearly defining it (8, 14, 18, 33), which renders their results somewhat difficult to interpret. We also note that conversation-analytical research has much to offer regarding the roles and mechanisms of mutual gaze (see for instance (3, 4)). In our own data set, instances of mutual gazing were found, but eye-contact could not be distinguished separately from mutual gaze since the distance between the musicians was too large.

More complex patterns that do not take place in dyadic interactions may also be investigated. Biasutti et al. (5) found instances of “multiple-direction eye contact”, i.e. gazing at more than one different musical partner in immediate succession. Research in non-musical domains on shared attention (16) allows us to distinguish an additional gaze pattern, hitherto unreported in music studies, namely the simultaneous gazing by two persons at a third. Our own data set showed that both gaze patterns occurred, however only rarely. We add that studying bodily communication *multimodally* would be a useful (and sought-after) way to advance the study of gaze patterning between partners. For instance, investigations into the mechanisms of interpersonal synchronisation could benefit from studying cueing gestures in relation to gaze (see for instance (6, 7)).

Our own research will proceed by studying the relationship between gaze and score characteristics. However, we foresee some challenges. First, the duration of the gazes at the partners may differ substantially: from as short as 40 ms (meaning that the cursor appeared twice in a row at more or less the same spot at an interval of 40 ms, which was the duration of a single frame during annotation) to around 2400 ms, although the mean duration for a partner-gaze was approximately 400 ms. Leaving aside that an explanation for such outliers would be interesting, long gazes have the ‘disadvantage’ that they cover a lot of the ongoing music and hence, become difficult to relate to just one moment in the ongoing stream of music. Furthermore, if a partner-gaze is at all related to a single moment in the score, it may precede or follow a score event. Alternatively, a gaze can be related to a passage of music without it being deliberately timed to precede or follow a certain moment within that passage. Indeed, the gaze patterning of a single musician may or may not change along contrasting sections in the music. Yet another scenario occurs when gazes occur quickly after one another, opening up the possibility that they may relate to the same moment in the music. This shows the complexity of gaze behaviour in our data set and indicates that data enabling access to the participants’ own processing of the score and their experience during playing would be valuable input for analysis. Thus, in addition to an analysis in relation to the score, we will analyse gaze in relation to the decision-making process during the rehearsal periods and the participants’ answers on the post-performance questionnaire. We hope that this will enable us to relate gaze to the musical task as perceived by the musicians and that this will prove a promising method to study the way gaze functions in ensemble playing. Meanwhile, the current study forms a complement to an artistic research project, in which the first author investigates gaze behaviour in her own trio (equally consisting of violin, clarinet and piano). It is expected that her self-tuition will be strengthened by the results from the current observational enquiry.

### Ethics and Conflict of Interes

The authors declare that the contents of the article are in agreement with the ethics described in http://biblio.unibe.ch/portale/elibrary/BOP/jemr/ethics.html and that there is no conflict of interest regarding the publication of this paper.

## References

[b1] Anantrasirichai N, Gilchrist ID, Bull DR Fixation identification for low-sample-rate mobile eye trackers. Proceedings of the IEEE International Conference on Image (ICIP 2016). 2016p. 3126-30. doi: 10.1109/ICIP.2016.7532935

[b2] Antonietti A., Cocomazzi D., & Iannello P. (2009). Looking at the audience improves music appreciation. Journal of Nonverbal Behavior, 33(2), 89–106. 10.1007/s10919-008-0062-x 0191-5886

[b3] Argyle M., & Cook M. (1976). Gaze and mutual gaze. Cambridge: Cambridge University press.

[b4] Bavelas J. B., Coates L., & Johnson T. (2002). Listener responses as a collaborative process: The role of gaze. Journal of Communication, 52(3), 566–580. 10.1093/joc/52.3.566 10.1093/joc/52.3.566 0021-9916

[b5] Biasutti M., Concina E., Wasley D., & Williamon A. (2016). Music regulators in two string quartet ensembles: A comparison of communicative behaviours between low- and high-stress performance conditions. Frontiers in Psychology, 7, 1229. 10.3389/fpsyg.2016.01229 1664-1078 27610089 PMC4997094

[b6] Bishop L, Goebl W Mapping visual attention of ensemble musicians during performance of “temporally-ambiguous” music. Paper presented at: Conference on Music & Eye-Tracking (MET17); 2017 8; Frankfurt, Germany.

[b7] Bishop L., & Goebl W. (2018). Beating time: How ensemble musicians’ cueing gestures communicate beat position and tempo. Psychology of Music, 46(1), 84–106. 10.1177/0305735617702971 0305-7356 29276332 PMC5718341

[b8] Blank M., & Davidson J. W. (2007). An exploration of the effects of musical and social factors in piano duo collaborations. Psychology of Music, 35(2), 231–248. 10.1177/0305735607070306 0305-7356

[b9] Brandler B. J., & Peynircioglu Z. F. (2015). A comparison of the efficacy of individual and collaborative music learning in ensemble rehearsals. Journal of Research in Music Education, 63(3), 281–297. 10.1177/0022429415597885 0022-4294

[b10] Dardard F., Gnecco G., & Glowinski D. (2016). Automatic classification of leading interactions in a string quartet. [TiiS] ACM Transactions on Interactive Intelligent Systems, 6(1), 1–27. 10.1145/2818739 2160-6455

[b11] Davidson J. W. (2012). Bodily movement and facial actions in expressive musical performance by solo and duo instrumentalists: Two distinctive case studies. Psychology of Music, 40(5), 595–633. 10.1177/0305735612449896 0305-7356

[b12] Davidson J. W., & Good J. M. M. (2002). Social and musical co-ordination between members of a string quartet: An exploratory study. Psychology of Music, 30(2), 186–201. 10.1177/0305735602302005 0305-7356

[b13] Duchowski A. (2007). Eye tracking methodology: theory and practice (2nd ed.). London: Springer-Verlag.

[b14] Ford L., & Davidson J. W. (2003). An investigation of members’ roles in wind quintets. Psychology of Music, 31(1), 53–74. 10.1177/0305735603031001323 0305-7356

[b15] Foulsham T., Cheng J. T., Tracy J. L., Henrich J., & Kingstone A. (2010). Gaze allocation in a dynamic situation: Effects of social status and speaking. Cognition, 117(3), 319–331. 10.1016/j.cognition.2010.09.003 0010-0277 20965502

[b16] Frischen A., Bayliss A. P., & Tipper S. P. (2007). Gaze cueing of attention: Visual attention, social cognition, and individual differences. Psychological Bulletin, 133(4), 694–724. 10.1037/0033-2909.133.4.694 0033-2909 17592962 PMC1950440

[b17] Fulford R., & Ginsborg J. (2014). Can you hear me? Effects of hearing impairments on verbal and non-verbal communication during collaborative musical performance. Psychology of Music, 42(6), 846–855. 10.1177/0305735614545196 0305-7356

[b18] Geeves A., McIlwain D. J., & Sutton J. (2014). The performative pleasure of imprecision: A diachronic study of entrainment in music performance. Frontiers in Human Neuroscience, 8(Oct), 863. 10.3389/fnhum.2014.00863 1662-5161 25400567 PMC4212675

[b19] Holler J., & Kendrick K. H. (2015). Unaddressed participants’ gaze in multi-person interaction: Optimizing recipiency. Frontiers in Psychology, 6, 98. 10.3389/fpsyg.2015.00098 1664-1078 25709592 PMC4321333

[b20] Jehoul A, Brône G, Feyaerts K Gaze patterns and fillers: empirical data on the difference between Dutch ‘euh’ and ‘euhm’. In: Paggio P, Navarretta C, editors Proceedings of the 4th European and 7th Nordic Symposium on Multimodal Communication (MMSYM 2016).Linköping: Linköping University Electronic Press;2017p. 43-50.

[b21] Kawase S. An exploratory study of gazing behavior during live performance. In: Louhivuori J, Eerola T, Saarikallio S, Himberg T, Eerola P, editors Proceedings of the 7th Triennial Conference of European Society for the Cognitive Sciences of Music (ESCOM); 2009 Jyväskylä: ESCOM 2009. p. 227-32.

[b22] Kawase S. (2014a). Assignment of leadership role changes performers’ gaze during piano duo performances. Ecological Psychology, 26(3), 198–215. 10.1080/10407413.2014.929477 1040-7413

[b23] Kawase S. (2014b). Gazing behavior and coordination during piano duo performance. Attention, Perception & Psychophysics, 76(2), 527–540. 10.3758/s13414-013-0568-0 1943-3921 24170378

[b24] Keller P. E., & Appel M. (2010). Individual differences, auditory imagery, and the coordination of body movements and sounds in musical ensembles. Music Perception, 28(1), 27–46. 10.1525/mp.2010.28.1.27 0730-7829

[b25] Kendon A. (1967). Some functions of gaze-direction in social interaction. Acta Psychologica, 26(1), 22–63. 10.1016/0001-6918(67)90005-4 0001-6918 6043092

[b26] King E., & Ginsborg J. (2011). Gestures and glances: interactions in ensemble rehearsal In Gritten A. & King E. (Eds.), New perspectives on music and gesture (pp. 177–201). Surrey: Ashgate.

[b27] Kurosawa K., & Davidson J. W. (2005). Nonverbal behaviours in popular music performance: A case study of The Corrs. Musicae Scientiae, 19(1), 111–136. 10.1177/102986490500900104 1029-8649

[b28] Liversedge S. P., Gilchrist I. D., & Everling S. (Eds.) (2011). The Oxfordhandbook of eye movements. Oxford: Oxford University Press 10.1093/oxfordhb/9780199539789.001.0001

[b29] Madell J., & Hébert S. (2008). Eye movements and music reading: Where do we look next? Music Perception, 26(2), 157–170. 10.1525/mp.2008.26.2.157 0730-7829

[b30] Moran N. Improvising musicians’ looking behaviours: duration constants in the attention patterns of duo performers. In: Demorest S, Morrison S, Campbell PS, editors Proceedings of the 11th International Conference on Music Perception and Cognition (ICMPC11); 2010 Seattle: ICMPC.

[b31] Morgan E., Gunes H., & Bryan-Kinns N. (2015a). The LuminUs: providing musicians with visual feedback on the gaze and body motion of their co-performers In Abascal J., Barbosa S., Fetter M., Gross T., Palanque P., & Winckler M. (Eds.), Proceedings, Part II: Human-Computer Interaction–INTERACT 2015 (pp. 47–54). Cham: Springer International Publishing; 10.1007/978-3-319-22668-2_4

[b32] Morgan E., Gunes H., & Bryan-Kinns N. (2015b). Using affective and behavioural sensors to explore aspects of collaborative music making. International Journal of Human-Computer Studies, 82, 31–47. 10.1016/j.ijhcs.2015.05.002 1071-5819

[b33] Pennill N, Timmers R Rehearsal processes and stage of performance preparation in chamber ensembles. Paper presented at: 25th Anniversary Edition of the European Society for the Cognitive Sciences of Music (ESCOM); 2017 8; Ghent, Belgium.

[b34] Puurtinen M. (2018). Eye on music reading: Amethodological review of studies from 1994 to 2017. Journal of Eye Movement Research, 11(2). 10.16910/jemr.11.2.2 1995-8692 PMC7725652 33828685

[b35] Rossano F. (2012). Gaze in conversation In Sidnell J. & Stivers T. (Eds.), The handbook of conversation analysis (pp. 308–329). Chichester: Wiley-Blackwell 10.1002/9781118325001.ch15

[b36] Silvey B. A. (2014). Strategies for improving rehearsal technique: Using research findings to promote better rehearsals. Update - University of South Carolina. Dept. of Music, 32(2), 11–17. 10.1177/8755123313502348 8755-1233

[b37] Stiefelhagen R. Tracking focus of attention in meetings. Proceedings of the 4th IEEE International Conference on Multimodal Interfaces. 2002 p. 273-80.doi: 10.1109/ICMI.2002.1167006

[b38] Vandemoortele S, De Beugher S, Brône G, Feyaerts K, Goedemé T, De Baets T, et al. Into the wild – Musical communication in ensemble playing. Discerning mutual and solitary gaze events in musical duos using mobile eye-tracking. Paper presented at: 2nd International Workshop on Vision and Eye Tracking in Natural Environments and Solutions & Algorithms for Gaze Analysis; 2015 9; Bielefeld, Germany.

[b39] Vandemoortele S., De Beugher S., Brône G., Feyaerts K., Goedemé T., De Baets T., Vervliet S. (2016). Into the Wild: Muzikale interactie in ensembles: een multimodale studie met eye-trackers. Leuven: Acco.

[b40] Vera B, Chew E, Healey PGT A study of ensemble synchronisationunder restricted line of sight. Proceedings of the International Conference on Music Information Retrieval; 2013; Curitiba, Brazilp. 293-98.

[b41] Williamon A., & Davidson J. W. (2002). Exploring co-performer communication. Musicae Scientiae, 6(1), 53–72. 10.1177/102986490200600103 1029-8649

[b42] Wittenburg P, Brugman H, Russel A, Klassmann A, Sloetjes H. ELAN: a professional framework for multimodality research. Proceedings of the 5th International Conference on Language Resources and Evaluation (LREC 2006).2006 p. 1556-9.

[b43] Yamada K, Ohgiri M, Furukawa T, Yuminaga H, Goto A, Kida N, et al. Visual behavior in a Japanese drum performance of Gion festival music. In: Duffy VG, editor. Digital Human Modeling. Applications in Health, Safety, Ergonomics and Risk Management: 5th International Conference, DHM 2014, Lecture Notes in Computer Science(Vol. 8529). Cham: Springer; 2014. p. 301-10.doi: 10.1007/978-3-319-07725-3_30

